# Genetic Correlation between Body Fat Percentage and Cardiorespiratory Fitness Suggests Common Genetic Etiology

**DOI:** 10.1371/journal.pone.0166738

**Published:** 2016-11-15

**Authors:** Theresia M. Schnurr, Anette P. Gjesing, Camilla H. Sandholt, Anna Jonsson, Yuvaraj Mahendran, Christian T. Have, Claus T. Ekstrøm, Anne-Louise Bjerregaard, Soren Brage, Daniel R. Witte, Marit E. Jørgensen, Mette Aadahl, Betina H. Thuesen, Allan Linneberg, Hans Eiberg, Oluf Pedersen, Niels Grarup, Tuomas O. Kilpeläinen, Torben Hansen

**Affiliations:** 1 Section of Metabolic Genetics, The Novo Nordisk Foundation Center for Basic Metabolic Research, Faculty of Health and Medical Sciences, University of Copenhagen, Copenhagen, Denmark; 2 Section of Biostatistics, Department of Public Health, Faculty of Health and Medical Sciences, University of Copenhagen, Copenhagen, Denmark; 3 Research Centre for Prevention and Health, The Capital Region of Denmark, Copenhagen, Denmark; 4 Department of Clinical Experimental Research, Rigshospitalet, Copenhagen, Denmark; 5 Department of Clinical Medicine, Faculty of Health and Medical Sciences, University of Copenhagen, Copenhagen, Denmark; 6 Department of Cellular and Molecular Medicine, Faculty of Health and Medical Sciences, University of Copenhagen, Copenhagen, Denmark; 7 Section of General Practice, Department of Public Health, Aarhus University, Aarhus, Denmark; 8 Medical Research Council Epidemiology Unit, University of Cambridge, Cambridge, United Kingdom; 9 National Institute of Public Health, University of Southern Denmark, Odense, Denmark; 10 Steno Diabetes Center, Gentofte, Denmark; INIA, SPAIN

## Abstract

**Objectives:**

It has long been discussed whether fitness or fatness is a more important determinant of health status. If the same genetic factors that promote body fat percentage (body fat%) are related to cardiorespiratory fitness (CRF), part of the concurrent associations with health outcomes could reflect a common genetic origin. In this study we aimed to 1) examine genetic correlations between body fat% and CRF; 2) determine whether CRF can be attributed to a genetic risk score (GRS) based on known body fat% increasing loci; and 3) examine whether the fat mass and obesity associated (*FTO*) locus associates with CRF.

**Methods:**

Genetic correlations based on pedigree information were examined in a family based cohort (n = 230 from 55 families). For the genetic association analyses, we examined two Danish population-based cohorts (n_total_ = 3206). The body fat% GRS was created by summing the alleles of twelve independent risk variants known to associate with body fat%. We assessed CRF as maximal oxygen uptake expressed in millilitres of oxygen uptake per kg of body mass (VO_2_max), per kg fat-free mass (VO_2_max_FFM_), or per kg fat mass (VO_2_max_FM_). All analyses were adjusted for age and sex, and when relevant, for body composition.

**Results:**

We found a significant negative genetic correlation between VO_2_max and body fat% (ρG = -0.72 (SE ±0.13)). The body fat% GRS associated with decreased VO_2_max (β = -0.15 mL/kg/min per allele, p = 0.0034, age and sex adjusted). The body fat%-increasing *FTO* allele was associated with a 0.42 mL/kg/min unit decrease in VO_2_max per allele (p = 0.0092, age and sex adjusted). Both associations were abolished after additional adjustment for body fat%. The fat% increasing GRS and *FTO* risk allele were associated with decreased VO_2_max_FM_ but not with VO_2_max_FFM_.

**Conclusions:**

Our findings suggest a shared genetic etiology between whole body fat% and CRF.

## Introduction

It has been discussed whether fitness or fatness is a more important determinant of health status. There is evidence that low cardiorespiratory fitness (CRF) and obesity are equally important predictors of mortality [1] and other health outcomes [2]. Furthermore, physical activity and high CRF are beneficial for health at any body weight [2, 3] and each of them may attenuate overweight and obesity-induced health risks [4].

CRF is commonly estimated by VO_2_max, a measure of the oxygen consumption during maximal exercise. Twin studies have shown that adiposity and CRF have strong genetic components, with heritability estimates of 50–90% for body-mass index (BMI) [5], 25–30% for body fat percentage (body fat%) [6] and 40–50% for CRF (VO_2_max) [7]. The link between development of obesity and level of physical fitness might be caused by a common genetic origin, rather than a causal effect.

Large-scale genome-wide association studies (GWAS) have identified more than one hundred loci associated with overall adiposity [8, 9], but no genetic variants are known to robustly associate with CRF. This may be due to insufficient sample sizes with data on CRF to identify variants with modest effects at the genome-wide significant level. GWAS have thus far identified twelve loci robustly associated with body fat percentage [9]. The strongest of these, *FTO*, was the first GWAS-identified susceptibility gene for common obesity [10]. Ever since, many studies have examined whether single nucleotide polymorphisms (SNPs) in the *FTO* loci are associated with lifestyle factors such as physical activity and other mediators leading to increased body weight [10]. While *FTO* does not seem to play a role in the regulation of physical activity levels [10], the relationship between *FTO* and obesity risk is modified by physical activity [11, 12]. There are, however, only few reports on *FTO* and physical fitness phenotypes. In a controlled exercise intervention study of 481 individuals, it was found that exercise-induced changes in adiposity were dependent on the *FTO* genotype [13]. In contrast, another study examining 846 young, healthy men failed to show that aerobic fitness in the untrained state is associated with the *FTO* genotype nor that it modifies the effect of *FTO* on body composition [14].

In the present study, we aimed to (1) examine genetic correlations between measures of adiposity and CRF in a family-based study sample; (2) determine whether inter-individual differences in CRF can be attributed to a genetic risk score (GRS) of GWAS-identified body fat% variants and whether GWAS-identified body fat% loci interact with CRF to modify levels of body fat%; (3) examine whether the *FTO* locus, known to exert the largest genetic effect on different adiposity measures, associates with CRF in two independent population-based cohorts of Danish ancestry.

## Materials and Methods

### Study populations

The Family cohort consists of 533 Danish individuals from 95 families with one parent suffering from type 2 diabetes and the other parent having no known diabetes. The families were identified and all non-diabetic family members (spouses, offspring and other relatives) were recruited through the outpatient clinic at the Steno Diabetes Center (Gentofte, Denmark) or through an ongoing family study at the University of Copenhagen (Copenhagen, Denmark) [15]. All participants of the Family cohort underwent measurement of height and weight. The amount of body fat was determined by bio-impedance (Biodynamics BIA 310e, H.A.W consulting, Denmark). Maximal oxygen intake (VO_2_max), was estimated from the heart rate response to a submaximal cycle ergometer exercise test with the Astrand-Rhyming nomogram [16]. We excluded family members if disagreement between questionnaire information on familial relationship and the actual genotypic resemblance was observed. Of the 435 individuals from families having four or more children, 230 with data on BMI, body fat% and CRF were included in the present genetic correlation analysis. The characteristics and relationships of the 230 individuals are shown in Table 1 and S1 Table.

**Table 1:** Clinical characteristics of three Danish study populations included into the analysis.

	Family cohort	ADDITION-PRO	Health2006
**n (f/m)**	230 (123/103)	716 (329/387)	2586 (1414/1172)
**Age, years**	39.4 (34; 42)	66.1 (60.9; 70.7)	49 (40; 59)
**BMI, kg/m**^**2**^	26.1 (4.5)	27.1 (4.4)	25.6 (4.3)
**Body fat percentage, %**	32.7 (10.3)	32.0 (8.1)	29.4 (8.8)
**Lean body mass, kg**	51.8 (10.7)	53.9 (11.3)	53.3 (11.2)
**VO**_**2**_**max, ml/kg/min**	32.6 (9.5)	29.8 (5.4)	31.9 (8.9)
**VO**_**2**_**max**_**FFM**_**, ml/kg FFM/min**	48.6 (11.2)	44.1 (7.6)	45.0 (10.6)
**VO**_**2**_**max**_**FM**_**, ml/kg FM/min**	122.6 (94.6)	94.7 (41.0)	126.5 (74.7)

Data in Table 1 are given as mean (standard deviation) or median (interquartile range). n: sample size, f: female, m: male, BMI: Body mass index, VO_2_max: maximal oxygen uptake scaled by body weight, VO_2_max_FFM_: maximal oxygen uptake scaled by fat free mass, VO_2_max_FM_: maximal oxygen uptake scaled by fat mass.

The ADDITION-PRO cohort is a population-based study of Danish individuals, aged 45–80 years at medium to high risk of developing type 2 diabetes, recruited during a stepwise screening procedure during 2001–2006. The screening procedure and the assessment of anthropometric measures, including BMI and body fat% for ADDITION-PRO have been described in detail elsewhere [17]. In short, height and weight, for the calculation of BMI, was measured in light indoor clothing and without shoes. Body fat% and weight were assessed by bioelectrical impedance using a leg-to-leg Tanita Body Composition Analyser (Tokyo, Japan). A subset of participants (n = 955) underwent an 8-min submaximal step test, during and after which heart rate was measured using a combined sensor (Actiheart, CamNTech Ltd., Cambridge, UK) [18]. The test was administered using the sensor manufacturer’s software to indicate the cycles of stepping up and down a 20.5-cm step bench; stepping frequency ranged from 15 to 33 step cycles per minute over the duration of the test [18]. The submaximal heart rate response to exercise load was modeled as linear [19] and extrapolated to age-predicted maximal heart rate [20] to estimate VO_2_max (Study characteristics in Table 1).

The Health2006 study is a population-based cohort consisting of a random sample of Danish men and women aged 18–69 years living in the southwestern part of the greater Copenhagen area [21]. Height and weight were measured wearing light clothes and no shoes. The amount of body fat was assessed by a Tanita Body Composition Analyzer (Illinois, USA)[21]. VO_2_max was estimated using the Danish step test according to instructions available at (www.health-calc.com/fitness-tests/the-danish-step-test). In short, the Danish step test is simple, requires little equipment and was developed for estimation of CRF in large epidemiological studies. The test is based on workload estimation of maximal oxygen uptake. It is a progressive test that starts with a stepping frequency of 0.2 steps/second which increases gradually until a maximal frequency of 0.8 steps/seconds at 6 minutes. VO_2_max (ml/min) was then calculated based on the height of the stepping bench, the duration of the test procedure, and the weight of the participant using a formula that has been validated against a Wattmax test [22]. Of the 2703 individuals that had been genotyped and underwent the submaximal step-test, two were excluded due to VO_2_max values below 10 mL/kg/min (Study characteristics in Table 1).

Prior to participation, informed written consent was obtained prior participation from all participants of the three studies described above. The Ethical Committee of Copenhagen (KA 93033 and KA 93033gm) approved the Family cohort study. The Ethical Committee of Copenhagen County (KA-20060011) and the Danish Data Protection Agency approved the Health2006 study. The Health2006 study was registered at www.clinicaltrials.gov (ClinicalTrials.gov identification number: NCT00316667, other study ID number: KA20060011). The ADDITION-PRO study was approved by the Scientifics Ethics Committee in the Central Denmark Region (20000183). All studies were conducted in accordance with the principles of the Declaration of Helsinki.

### Genotyping

#### ADDITION-PRO

Participants of the ADDITION-PRO cohort (n = 1657) were genotyped by the Illumina Infinium HumanCoreExome Beadchip platform (Illumina, San Diego, CA). Genotypes were called using the Genotyping module (version 1.9.4) of GenomeStudio software (version 2011.1, Illumina). We excluded 109 closely related individuals, individuals with extreme inbreeding coefficients, individuals with mislabelled sex, individuals with a call rate <95%, duplicates and individuals identified as ethnic non-European outliers, leaving 1548 individuals who passed all quality control criteria. Additional genotypes were imputed into 1000 genomes phase 1 [23] using impute2 [24]. The imputation quality was high (proper_infor > 0.95) for all imputed variants included in the current study. All variants obeyed Hardy Weinberg equilibrium (p > 0.05).

#### Health2006

Participants from the Health2006 (n = 2883) cohorts were genotyped by Metabochip on the Illumina HiScan platform (Illumina, San Diego, CA, USA). Genotypes were called using the GenomeStudio software (version 2011.1, Illumina). We excluded individuals with low call rate, mislabeled sex, relatedness, extreme inbreeding coefficient and with a high discordance rate to previously genotyped SNPs, leaving 2804 individuals for whom genotyping was successful accomplished. All variants obeyed Hardy Weinberg equilibrium (p > 0.05).

### Genetic correlation and GRS analyses

#### Genetic and environmental correlations

Genetic, phenotypic and environmental correlations in the Family cohort were calculated using SOLAR (http://solar.txbiomedgenetics.org, version 4.2.0) [25]. The additive effect of shared genes was calculated as described elsewhere [26]; individuals belonging to the same family were assumed to be sharing the same household. Neither body fat% nor CRF were significantly affected by the shared environment and thus it was not included in the genetic correlation analysis. We tested whether the genetic correlation is significantly different from complete genetic correlation (P_different from 1_) or from no correlation (P_different from 0_).

#### GRS construction and association analyses

Genotypes were coded according to the number of body fat% increasing alleles based on 12 independent variants shown to be associated with body fat% in a large-scale GWAS meta-analysis [9]. We constructed a weighted GRS by summing the number of body fat%- increasing alleles weighted by the effect size of the variant estimated in the GWAS discovery study [9].

In the discovery cohort ADDITION-PRO, all genotypes were retrieved from the imputed dataset and genetic risk scores were calculated based on dosage information. Of the 955 individuals with information on step-test derived VO_2_max, n = 716 individuals had valid genotype information and were included in the subsequent analyses. For the *FTO* association and interaction analyses, the *FTO* rs1558902 variant was directly genotyped in all 716 individuals and therefore not retrieved from dosage information (S2 Table).

In Health2006, seven of the twelve GWAS identified body fat% SNPs were present on the Metabochip (rs1558902, rs6567160, rs6755502, rs693839, rs543874, rs3761445, rs757318), three SNPs (rs2943646, rs7609045, rs7187776) were captured by perfect proxies (r^2^ = 1), one (rs4794018) was captured by a proxy with r^2^ = 0.93 and one SNP (rs6857) was not covered by the Metabochip. Proxy search was performed using 1000 Genomes Pilot 1 data to estimate linkage disequilibrium using the SNP annotation proxy search tool (SNAP, http://www.broadinstitute.org/mpg/snap)[27]. Hence, a total of 11 SNPs for Health2006 are included in the GRS. Of the 2586 participants that had information on both genotypes and VO_2_max, 96 were excluded due to missing genotype information on ≥ 1 SNP. This allowed us to include a total of 2490 individuals into the GRS analysis. Data on *FTO* was available for all but two of the 2586 individuals; these were included into the *FTO* association and interaction analysis (S2 Table).

### Statistical analyses

Analyses in ADDITION-PRO and Health2006 were performed using R software (version 3.2.0, The R Foundation for Statistical Computing, Boston, MA, USA). For our analysis we expressed CRF relative to body weight, fat-free mass (FFM) and fat mass (FM), denoted VO_2_max (ml/kg/min), VO_2_max_FFM_ (ml/kg FFM/min) and VO_2_max_FM_ (ml/kg FM/min). After Bonferroni correction for multiple testing for the three traits tested, p<0.017 was considered statistically significant. Associations between the GRS and CRF as well as between *FTO* rs1558902 and CRF were examined by linear regression using additive genetic models. Analyses were adjusted for age and sex or age, sex and body composition (BMI, body fat%, FFM, FM). Linear regression models including an interaction term were used to test gene × CRF interactions on body fat%, adjusted for age and sex. All association analyses were first performed in ADDITION-PRO and then replicated in Health2006. Subsequently, we combined the effect size estimates and standard errors (SE) derived from the linear regression analyses using fixed effects meta-analyses, including a total of 3206 individuals (n_Health2006_ = 2490, n_ADDITION-PRO_ = 716) for the GRS analysis and 3300 individuals (n_Health2006_ = 2584, n_ADDITION-PRO_ = 716) for the *FTO* analysis using the ‘meta’ package (version 4.2–0) for R. We focus the following sections of this manuscript on the results from the meta-analysis. All cohort specific results for the associations are shown in S3 Table (Genetic risk score associations) and S4 Table (*FTO* associations).

## Results

### Heritability estimates and genetic correlations

Within the Family cohort, we found that additive genetic effects (*h*^*2*^) explained 25% (SE 15%) of the variation in BMI, 53% (SE 12%) of the variation in body fat%, 48% (SE 12%) of the variation in fat mass (kg) and 41% (SE 11%) of the variation in fat-free body mass (kg). We found that additive genetic effects explained 49% (SE 15%) of the variation in CRF relative to body weight (VO_2_max), 37% (SE 15%) of the variation in CRF relative to fat-free mass (VO_2_max_FFM_) and 57% (SE 15%) in CRF relative to fat mass (VO_2_max_FM_). All heritability estimates were adjusted for age and sex (S5 Table).

We calculated genetic (ρG) and phenotypic (ρP) correlations between VO_2_max and measures of body composition such as BMI, lean body fat (kg), fat mass (kg) and body fat%. Significant genetic correlations between VO_2_max and body fat% (ρG = -0.72 (SE ±0.13); p_different from 0_ < 0.01, p_different from 1_ < 0.01) and absolute VO_2_ (ml/min) and fat mass (kg) (ρG = -0.70 (SE ±0.17); p_different from 0_ < 0.01, p_different from 1_ < 0.01) were found. No genetic correlations were found between VO_2_max and BMI (ρG = -0.28 (SE ±0.22); p_different from 0_ = 0.24, p_different from 1_ < 0.01) nor VO_2_max and lean body mass (kg) (ρG = 0.39 (SE ±0.26); p_different from 0_ = 0.13, p_different from 1_ = 0.01).

### Genetic risk score association and interaction analysis

To examine whether genetic variants known to associate with body fat% contribute to the significant genetic correlation between VO_2_max and body fat%, we tested for associations between CRF and a GRS for body fat%, the latter constructed of the 12 known GWAS identified variants. The body fat% GRS ranged from 2 to 18 risk alleles (median = 10). The GRS was associated with body fat% (β = 0.22% per allele; p < 0.0001; age and sex adjusted) and showed an inverse association with VO_2_max (β = -0.15 mL/kg/min per additional risk allele, p = 0.0034). The association was abolished after additional adjustment for body fat% (β = -0.05, p = 0.27), indicating that about two thirds of the association was mediated by adiposity. We also found that the GRS was associated with VO_2_max_FM_ but not with VO_2_max_FFM_ (Table 2).

**Table 2:** Associations between body fat% GRS and CRF expressed in VO_2_max (ml/kg/min), VO_2_max_FFM_ (ml/kg FFM/min) as well as VO_2_max_FM_ (ml/kg FM/min) after meta-analysis of two Danish cohorts (ADDITION-PRO (n = 716) and Health2006 (n = 2490)).

Trait	Covariates	Per allele effect size on CRF (95% CI)	P
**VO**_**2**_**max (ml/kg/min)**	age, sex	-0.15 (-0.26 to -0.05)	0.0034
**VO**_**2**_**max (ml/kg/min)**	age, sex, body fat%	-0.05 (-0.15 to 0.04)	0.27
**VO**_**2**_**max (ml/kg/min)**	age, sex, fat-free mass	-0.11 (-0.21 to -0.0006)	0.04
**VO**_**2**_**max**_**FFM**_ **(ml/kg FFM/ min)**	age, sex	-0.06 (-0.19 to 0.07)	0.38
**VO**_**2**_**max**_**FFM**_ **(ml/kg FFM/min)**	age, sex, body fat%	-0.07 (-0.20 to 0.06)	0.30
**VO**_**2**_**max**_**FM**_ **(ml/kg FM/ min)**	age, sex	-0.01 (-0.02 to -0.007)	< 0.0001
**VO**_**2**_**max**_**FM**_ **(ml/kg FM/min)**	age, sex, fat-free mass	-0.008 (-0.01 to -0.003)	0.0033

Associations between body fat% GRS and CRF were examined by linear regression using additive genetic models. Data for VO2maxFM was log-transformed. Models were adjusted for age and sex or age, sex and body composition. Cohort specific association results can be found in S3 Table.

**Fig 1:**
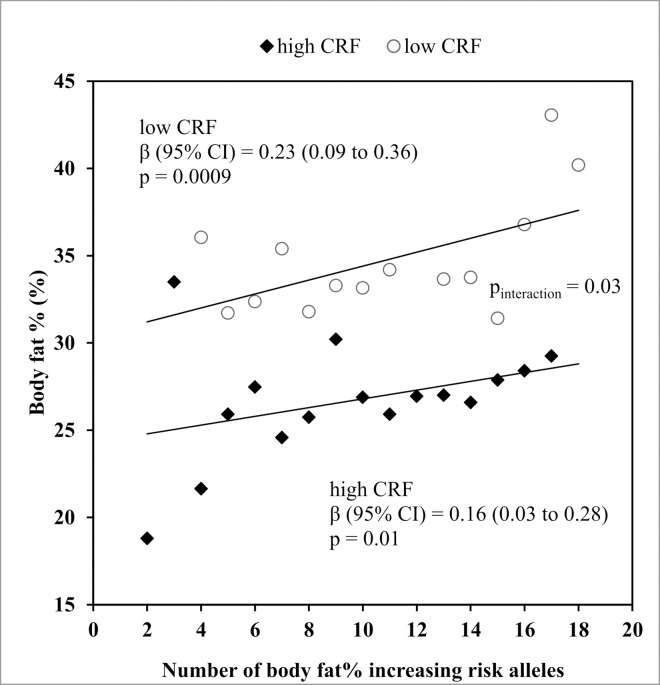
Association between the body fat% GRS and body fat% in the low CRF and high CRF groups after meta-analysis of two Danish cohorts (ADDITION-PRO and Health2006, a total n = 3206). Weighted mean body fat% for each number of body fat% risk alleles was calculated based on the relative weight for each cohort. CRF was objectively measured by step test and the study participants of both cohorts were stratified by the respective median of VO_2_max to form “high CRF” and “low CRF” groups. We tested for body fat% GRS x CRF interaction effects using linear models (P_interaction_ = 0.03).

After stratifying the study participants of both cohorts by the respective median of VO_2_max to form “high CRF” and “low CRF” groups, we demonstrated that the magnitude of the effect of each additional body fat% increasing risk allele was 30% smaller in individuals with high CRF (β = 0.16 kg/m^2^) than in individuals with low CRF (β = 0.23 kg/m^2^). The test of interaction between CRF and body fat% GRS on body fat% showed the expected direction of effect but was not statistically significant after adjustment for multiple testing (β_interaction_ = -0.01, p = 0.03) (Fig 1).

### FTO association analysis

The minor *FTO* rs1558902 A allele was associated with higher body fat% (β = 0.62 kg/m^2^; p < 0.0001; age and sex adjusted) and we found that VO_2_max associated with body fat% (p < 0.0001). The body fat%-increasing *FTO* allele was associated with a 0.42 mL/kg/min unit lower VO_2_max per allele (p = 0.009, adjusted for age and sex). The association was abolished when additional adjustment for BMI (p = 0.58) was done. *FTO* was also significantly associated with VO_2_max_FM_ but not with VO_2_max_FFM_ (Table 3).

**Table 3:** Associations between the *FTO* rs1558902 A-allele and VO_2_max (ml/kg/min), VO_2_max_FM_ (ml/kg FFM/min) as well as VO_2_max_FFM_ (ml/kg FM/min) after meta-analysis of two Danish cohorts (ADDITION-PRO (n = 716) and Health2006 (n = 2586)), assuming a fixed effect model.

	Covariates	Effect	SE (range)	P
**VO**_**2**_**max (ml/kg/min)**	mage, sex	-0.42	(-0.74 to -0.09)	0.0092
**VO**_**2**_**max (ml/kg/min)**	age, sex, BMI	-0.11	(-0.40 to 0.18)	0.46
**VO**_**2**_**max (ml/kg/min)**	age, sex, fat-free mass	-0.22	(-0.54 to 0.09)	0.17
**VO**_**2**_**max**_**FFM**_ **(ml/kg FFM/min)**	age, sex	-0.12	(-0.53 to 0.30)	0.58
**VO**_**2**_**max**_**FFM**_ **(ml/kg FFM/min)**	age, sex, BMI	-0.07	(-0.47 to 0.34)	0.74
**VO**_**2**_**max**_**FFM**_ **(ml/kg FFM/min)**	age, sex, fat- mass	-0.004	(-0.02 to 0.16)	0.68
**VO**_**2**_**max**_**FM**_ **(ml/kg FM/min)**	age, sex	-0.03	(-0.05 to -0.01)	0.00040
**VO**_**2**_**max**_**FM**_ **(ml/kg FM/min)**	age, sex, BMI	-0.0019	(-0.01 to 0.010)	0.76
**VO**_**2**_**max**_**FM**_ **(ml/kg FM/min)**	age, sex, fat-free mass	-0.01	(-0.03 to 0.002)	0.080

Associations between of the A- allele of *FTO* rs1558902 and CRF, expressed in VO_2_max (ml/kg/min), VO_2_max_FFM_ (ml/kg FFM/min) as well as VO_2_max_FM_ (ml/kg FM/min), were examined by linear regression assuming additive genetic models. Data for VO_2_max_FM_ was log-transformed. Models were adjusted for age and sex or age, sex and body composition. Study characteristics of ADDITION-PRO (n = 716) and Health2006 (n = 2586) according to *FTO* rs1558902 genotype can be found in S6 Table. Cohort specific association results can be found in S4 Table.

## Discussion

In the present study, we found that additive genetic effects explain part of the variation in CRF and exposed a pleiotropic effect between genes contributing to VO_2_max and body fat, both when measured as % and in kg. A GRS based on 12 validated body fat% increasing genetic variants identified through GWAS, and the minor *FTO* rs1558902 allele alone, showed an association with decreased VO_2_max that was mediated by adiposity. Lastly, the interaction of VO_2_max × body fat%-associated GRS on body fat% showed the expected, direction of effect where fitness attenuated the effect of body fat%-associated loci on body fat%, but this result was non-significant after adjustment for multiple testing.

The estimated genetic component of BMI, body fat% and VO_2_max was within the range of previous reports [5–7]. We added another dimension to our study, namely expressing VO_2_max relative to fat-free mass (VO_2_max_FFM_) and fat mass (VO_2_max_FM_). While there are reports concluding that VO_2_max relative to fat-free mass is truly independent of adiposity and the best indirect estimate of metabolic capacity of the skeletal muscles [28, 29], we also calculated VO_2_max relative to fat mass to be able to distinguish between CRF scaled by body weight, fat-free mass and fat-mass. We found that VO_2_max_FM_ is more heritable compared to VO_2_max_FFM_.

We found a significant negative genetic correlation between body fat (expressed in kg as well as %) and CRF but not between BMI or lean body mass and CRF. Body fat% is a more accurate measure of adiposity than is BMI. Body fat% distinguishes between fat mass and lean body mass, whereas BMI is a composite measure of fat mass and lean mass. Further, lean body mass is thought to be the body compartment explaining the major influence of body weight on VO_2_max[28]; yet, we did not find any pleiotropic genetic effects between VO_2_max (ml/kg/min) and lean body mass. The identified genetic correlation is indicative of shared genetics between the two traits (body fat% and VO_2_max) and may exist due to pleiotropy between both traits, meaning that the same genes are influencing both phenotypic traits or a pathway common for the phenotypes or due to gene-gene interactions, where the impact of one gene is influenced by another (set of) gene(s). We find that both scenarios are equally intriguing as these results are suggestive of a crosstalk between adipose tissue and the ability to consume oxygen in the lean body compartment, knowing that oxygen uptake in fat tissue is negligible.

In a comprehensive twin-study (n = 756 complete twin pairs) that explored genetic and environmental correlations between CRF and adiposity, it appeared that genetic, environmental as well as phenotypic influences linked greater CRF to lower levels of adiposity [30]. The authors of the same study proposed that there may be some genetic variants that contribute both to the propensity to develop adiposity and a preference for a sedentary lifestyle, resulting in reduced CRF, or resistance to formation of adiposity and preference for being physically active, resulting in an increased CRF [30]. Moreover, physical fitness and adiposity are related to inflammatory markers (such as cytokines, adipokines, C-reactive proteins, etc.) and it has been suggested that it may not be adiposity *per se* that mediates the association between fitness and inflammation status but instead that both, CRF and adiposity, may share the same causal pathways [31]. A further potential mechanism regulating body fat% and CRF through a common set of genes may be mediated via hypothalamic brain regions. At least three of the known body fat% increasing SNPs: *FTO[8]*, *TMEM18[8]* and *CRTC1[9]* are thought to be expressed in hypothalamic brain regions, the brain region that regulates energy homeostasis. Studies in animals have shown that the control of voluntary movement, and therefore potentially CRF, resides in similar central neural pathways as energy intake [32, 33]. The central nervous system could therefore be an upstream region where body fat% increasing SNPs share a common biological influence on both adiposity and physical fitness phenotypes.

We then tested the effects of body fat% GRS on CRF in a meta-analysis of two Danish cohorts and demonstrated that each additional risk allele was associated with a 0.14 mL/min/kg decrease in VO_2_max, explaining 0.06% of the variance in VO_2_max in ADDITION-PRO and 0.2% in Health2006. We tested whether VO_2_max_FM_ and VO_2_max_FFM_ affect the body fat% GRS association differently and found that scaling CRF by fat-free mass abolished the association, whereas scaling CRF by fat mass seemed to signify our results (also after controlling for fat-free mass) as compared with the traditional scaling by body mass. Therefore, variants known to associate with body fat% may have no direct influence on CRF but expressing CRF relative to body mass or fat mass, both heavily influenced by adiposity, may cause the associations. Our approach to investigate the genetic overlap between body fat% and CRF in testing for associations between adiposity SNPs and CRF is inconclusive. Therefore, we suggest applying more refined techniques such as large-scale multivariate GWAS analysis in future studies, to be able to define the common set of genes that we hypothesize to be regulating oxygen uptake in muscle and body fat% simultaneously.

While we found an adiposity mediated association between CRF and body fat% GRS, it is unlikely that all of the gene variants comprising this GRS contribute to this effect. Individually, we tested whether *FTO*, the strongest known susceptibility locus for common obesity associates with VO_2_max and we found an inverse, adiposity-mediated association. The obesity risk gene *FTO* encodes a 2-oxoglutarate-dependent nucleic acid demethylase [34, 35], that is expressed in several peripheral tissues as well as in brain regions affecting energy balance [10, 36]. While previous studies tried to uncover a link between *FTO* and appetite regulation and the propensity to exercise controlled by the brain (reviewed in [10]), recent emerging data now points to a peripheral role for risk variants in the *FTO* locus in a pathway that is regulating adipocyte metabolism and controlling energy storage and energy dissipation [37, 38].

Participants of the ADDITION-PRO study were recruited by a stepwise screening procedure that identified Danes at a medium-to-high-risk scale of developing diabetes versus the Health2006 study population consisted of a randomly selected general population sample recruited through the Civil registration system from inhabitants in eleven municipalities in the Capital Region of Denmark. Individuals in ADDITION-PRO were overall less fit and older as compared to individuals in Health2006 and as such the two studies are not fully comparable. We speculate that the difference in age, health status and overall physical fitness across the cohorts could have diminished the effect of genetic variants known to associate with body fat% on VO_2_max therefore the effect may penetrate stronger in ADDITION-PRO. Our speculation of observed cohort differences due to physical fitness levels are in line with the study set up by Huuskonen et al. of healthy young males that did not show an association between the *FTO* obesity-linked variant and CRF. The cross-sectional study included 846 healthy young males and CRF was objectively assessed by a maximal bicycle ergometer test [14]. Even though a trend was observed, it is very likely that any effects of *FTO* variants on CRF was diminished by insufficient statistical power or overall fairly high baseline physical fitness levels throughout the study population. Ultimately, our findings are only generalizable to other populations with similar characteristics and therefore, our findings need replication in other independent study populations. We are aware that other factors that we could not control for in our analysis may have effects on estimates of CRF. We estimated that 49% of variation in CRF is heritable, but other determinants such as physical activity, dietary intake, disease status, biochemical measures, body composition and age are found to account for the remaining variation in CRF [39].

In ADDITION-PRO and Health2006, CRF was estimated by a submaximal step test [17, 21]. While submaximal tests do not provide accurate estimates of CRF at the individual level, the estimates are valid for studies in large populations. In a validation study, the correlation between the Danish step test and a maximal test of cardiorespiratory fitness was moderate to high, 0.77 in women and 0.69 in men, and the authors suggested the use of the Danish step test as a safe and feasible alternative to the more time-consuming watt-max test as a method for estimation of VO_2max_ in large population-based studies [22]. Nonetheless, it is important to replicate our observations using direct measures of CRF in future studies.

In conclusion, our results suggest a shared genetic etiology between body fat% and CRF. If replicated in future studies, ideally with direct measures of maximal oxygen consumption and more accurate measures of body fat% such as DEXA, our findings suggest that fat mass may have an indirect effect on maximal oxygen uptake. However, polymorphisms known to associate with body fat% could not explain the shared genetics independent of adiposity with *FTO* having a potential pleiotropic effect.

## Supporting Information

S1 Table Relationships of relative pairs within the Family cohort, included in the genetic correlation analysis. (XLSX)

S2 Table Overview of SNPs included into the fat%- GRS association analysis. An overview of the SNPs presented in Lu et al. and the SNPs investigated in the population-based Health2006 and ADDITION-PRO cohort and proxy (r2) when included in the analysis for Health2006. *Fat% increasing allele, EAFs (effect allele frequencies), other allele and effect sizes from the SNPs reported by Lu et al. 2015 for the sex-combined European-ancestry GWAS + ExomeChip analysis [9].
(XLSX)

S3 Table Associations between body fat% GRS and VO_2_max (ml/kg/min) as well as VO_2_max_FFM_ (ml/kg FFM/min) and VO_2_max_FM_ (ml/kg FM/min) in ADDITION-PRO (n = 716), Health2006 (n = 2490) assuming a fixed effect model. Associations between BMI and body fat% GRS and CRF were examined by linear regression using additive genetic models. Data for VO_2_max_FM_ was log-transformed. Models were adjusted for age and sex or age, sex and body composition.
(XLSX)

S4 Table Associations between the *FTO* rs1558902 and VO_2_max (ml/kg/min), VO_2_max_FFM_ (ml/kg FFM/min) as well as VO_2_max_FM_ (ml/kg FM/min) in ADDITION-PRO and Health2006, assuming a fixed effect model. Data are given in means ± standard error (SE) and median (interquartile ranges). Associations between *FTO*-rs1558902 and CRF were examined by linear regression using additive genetic models. VO_2_max_FM_ was log-transformed. Models were adjusted for age and sex or age, sex and body composition.
(XLSX)

S5 Table Results from the ACE models for calculation of the heritability on measures of body composition and CRF within the Family cohort, adjusted for sex and age. **ACE modelling is used to indicate what proportion of variance in a trait is contributed to its additive genetic effects (h**^**2**^**).** h^2^ (SE) = additive genetic effect. FM = fat mass, FFM = fat-free mass.
(XLSX)

S6 Table Study characteristics of ADDITION-PRO (n = 716) and Health2006 (n = 2586) according to *FTO* rs1558902 genotype. Data are given in means ± standard error (SE) and median (interquartile ranges).
(XLSX)
